# Anthropogenic events and responses to environmental stress are shaping the genomes of Ethiopian indigenous goats

**DOI:** 10.1038/s41598-024-65303-x

**Published:** 2024-06-28

**Authors:** Shumuye Belay, Gurja Belay, Helen Nigussie, Abulgasim M. Ahbara, Abdulfatai Tijjani, Tadelle Dessie, Getinet M. Tarekegn, Han Jian-Lin, Siobhan Mor, Helina S. Woldekiros, Keith Dobney, Ophelie Lebrasseur, Olivier Hanotte, Joram M. Mwacharo

**Affiliations:** 1 Tigray Agricultural Research Institute, Mekelle, Ethiopia; 2 Department of Microbial, Cellular and Molecular Biology, Addis Ababa University, Addis Ababa, Ethiopia; 3 Animal and Veterinary Sciences, Scotland’s Rural College (SRUC), Roslin Institute Building, Midlothian, UK; 4 Department of Zoology, Misurata University, Misurata, Libya; 5 The Jackson Laboratory, Bar Harbor, ME 04609 USA; 6 International Livestock Research Institute (ILRI), Addis Ababa, Ethiopia; 7 Institute of Biotechnology (IoB), Addis Ababa University, Addis Ababa, Ethiopia; 8 CAAS-ILRI Joint Laboratory on Livestock and Forage Genetic Resources, Beijing, China; 9 Institute of Animal Science, Chinese Academy of Agricultural Sciences (CAAS), Beijing, China; 10 Institute of Infection, Veterinary and Ecological Sciences, University of Liverpool, Liverpool, UK; 11 Department of Anthropology, Washington University in St. Louis, St. Louis, USA; 12 Department of Archaeology, Classics and Egyptology, University of Liverpool, Liverpool, UK; 13 University of Sydney, Sydney, Australia; 14 Palaeogenomics and Bioarchaeology Research Network, School of Archaeology, University of Oxford, Oxford, UK; 15 School of Life Sciences, University of Nottingham, Nottingham, UK; 16 Small Ruminant Genomics, International Centre for Agricultural Research in the Dry Areas (ICARDA), Addis Ababa, Ethiopia

**Keywords:** Genetics, Molecular biology

## Abstract

Anthropological and biophysical processes have shaped livestock genomes over Millenia and can explain their current geographic distribution and genetic divergence. We analyzed 57 Ethiopian indigenous domestic goat genomes alongside 67 equivalents of east, west, and north-west African, European, South Asian, Middle East, and wild Bezoar goats. Cluster, ADMIXTURE (K = 4) and phylogenetic analysis revealed four genetic groups comprising African, European, South Asian, and wild Bezoar goats. The Middle Eastern goats had an admixed genome of these four genetic groups. At K = 5, the West African Dwarf and Moroccan goats were separated from East African goats demonstrating a likely historical legacy of goat arrival and dispersal into Africa via the coastal Mediterranean Sea and the Horn of Africa. F_ST_, XP-EHH, and H*p* analysis revealed signatures of selection in Ethiopian goats overlaying genes for thermo-sensitivity, oxidative stress response, high-altitude hypoxic adaptation, reproductive fitness, pathogen defence, immunity, pigmentation, DNA repair, modulation of renal function and integrated fluid and electrolyte homeostasis. Notable examples include *TRPV1* (a nociception gene); *PTPMT1* (a critical hypoxia survival gene); *RETREG* (a regulator of reticulophagy during starvation), and *WNK4* (a molecular switch for osmoregulation). These results suggest that human-mediated translocations and adaptation to contrasting environments are shaping indigenous African goat genomes.

## Introduction

The domestication of goats (*Capra hircus*) occurred in the Fertile Crescent ~ 11,000 years ago from a mosaic of wild Bezoar (*Capra aegagrus*)^1–3^, ushered in one of the key milestones in the shift from hunting and gathering to the beginnings of sedentary agriculture. Following their domestication, goats demonstrated remarkable versatility in integrating and adapting to novel environments during their human-mediated dispersal occurring throughout the subsequent millenia^4^. Over time, their economic significance increased, and in recent times, rising demand for animal products has driven genetic-merit-based selective breeding for elite dairy (e.g., Alpine), meat (e.g., Boer), and fibre (e.g., Angora) landraces, along with other breeds with unique characteristics (https://www.fao.org/dad-is/browse-by-country-and-species/en/). In contrast, natural selection has given rise to significant variation among indigenous varieties that have remained relatively nondescript in genotype and phenotype. Though less productive than elite breeds under an intensive production system, indigenous breeds carry important alleles and/or allelic combinations that confer adaptive plasticity to local environments.

Africa has a complex topography that influences agro-eco-climatic patterns and adaptive variation in domestic and wild species. These agro-eco-climates are well represented in Ethiopia which is home to a broad suite of indigenous domestic goat landraces that are uniquely adapted to diverse environments^5^. Domestic goats have a long history in the continent and in particular the Horn of Africa. For instance, the earliest occurrence of goats in the Ethiopian highlands and Sudanese Nile dates to the late 6th to early 5th millennia BC as attested by zooarchaeological findings^6,7^. Furthermore, increasing evidence indicates that north-east Africa (including the Red Sea area) and the Horn of Africa were a key arena for early socio-cultural and commercial exchange between mobile pastoralist groups from beyond the region^8^. A study of indigenous goats from the Horn could thus shed light on the long term dynamics of livestock-human interrelationships.

The Genome diversity of local goats in northeast Africa and the Horn has not been extensively studied. Investigations done so far, using mitochondrial DNA^9–11^, microsatellites^12,13^, and SNP genotypes^14,15^ indicate high genetic diversity with little or no phylogeographic structure. However, mtDNA assesses only maternal divergence, microsatellites suffer low genome coverage, and SNP microarrays are prone to ascertainment bias compared to whole-genome resequencing data^16^. The only whole-genome study^17^ undertaken to date analysed selection signatures in two out of 12 Ethiopian indigenous goat populations, leaving a major portion of the genomic variation poorly explored or investigated and therefore limiting interpretations at the local and global level. We therefore generated and analysed 57 whole-genomes from 12 Ethiopian indigenous domestic goat populations. The data were combined with previously sequenced genomes of 67 goats from east, west, and north-west Africa, Europe, South Asia, and the Middle East (Iran) and wild Bezoar goats. This analysis allowed us to: (1) characterise the current genomic landscape of Ethiopian indigenous goats vis a vis the non-Ethiopian ones, (2) describe a refined picture of the genetic and demographic history of Ethiopian indigenous goats in the wider context of other African and non-African goat populations, and (3) detect genome-wide signatures of positive selection for local adaptation.

## Results

### Sequence mapping quality and variant discovery

Whole-genome sequencing of the 57 Ethiopian goats yielded 2.8 Gb of paired-end raw reads (420 Gb) with a length of 150 bp. On average, 233.96 (218.84–249.22) Mb of clean reads were obtained per population. More than 99.75% of the clean reads mapped against the ARS1 goat reference genome assembly (GenBank accession number GCA_001704415.1) with a coverage of ~ 99.59%. These mapped reads generated an average sequencing depth of 9.71-fold (Supplementary Table S3). A total of 24,759,579 high-quality SNPs were identified resulting in a mean SNP density of 6.18 ± 4.92/Kb (Supplementary Tables S2 and S3). Validation of the SNPs on the *C. hircus* dbSNP reference panel showed that 29.73%, 0.81%, and 40.12% were novel, exonic, and intergenic, respectively (Supplementary Table S2, Supplementary Figs. S1, S2).

### Genome-wide genetic diversity and population structure

The mean values of the genetic diversity indices are shown in Table 1. Among Ethiopian goats, the mean and standard deviation of H_O_, H_E_, π, and D_ST_ were 0.347, 0.371, 0.0021, and 0.303, respectively. The H_O_, H_E,_ and D_ST_ were highest in Ethiopian goats. The F_HOM_ and F_RoH_ averaged 0.064 ± 0.04 and 0.059 ± 0.01, respectively in Ethiopian goats; the highest values were in Italian (F_HOM_ = 0.246 ± 0.16) and Pakistan (F_HOM_ = 0.243 ± 0.11) goats. In Ethiopian goats, the mean number and length of RoH were 932.9 ± 90.31 and 174.14 ± 36.31 Mb, respectively; the highest values were in Pakistan goats and averaged 1663.4 ± 811.17 and 410.27 ± 233.37 Mb, respectively.

**Table 1:** Indicators of genetic diversity and variation in the 25 Ethiopian and non-Ethiopian goat populations analysed in the study.

Population	N	Π	H_O_	H_E_	D_ST_	RoH number	RoH Length (Mb)	F_HOM_	F_RoH_
		Mean	Mean	Mean	Mean	Mean ± SD	Mean ± SD	Mean ± SD	Mean ± SD
Abergelle	4	0.002	0.365	0.392	0.319	966 ± 72	184 ± 50.4	0.068 ± 0.03	0.063 ± 0.02
Afar	5	0.002	0.331	0.354	0.293	846 ± 142	162 ± 61.7	0.065 ± 0.04	0.056 ± 0.02
Agew	5	0.002	0.319	0.362	0.303	1004 ± 127	207 ± 66.8	0.117 ± 0.16	0.071 ± 0.02
Ambo	5	0.002	0.363	0.391	0.319	974 ± 123	200 ± 59.1	0.072 ± 0.03	0.068 ± 0.02
Arsi-Bale	5	0.002	0.339	0.359	0.290	958 ± 100	193 ± 41.6	0.056 ± 0.04	0.066 ± 0.01
Gonder	5	0.002	0.415	0.442	0.355	909 ± 25.8	155 ± 3.0	0.061 ± 0.02	0.053 ± 0.00
Gumuz	5	0.002	0.345	0.361	0.291	929 ± 54.8	171 ± 17.9	0.045 ± 0.02	0.059 ± 0.01
HHG	5	0.002	0.332	0.354	0.292	793 ± 32.4	131 ± 10.7	0.060 ± 0.03	0.045 ± 0.00
Keffa	5	0.002	0.344	0.363	0.293	1044 ± 65.4	199 ± 24.2	0.054 ± 0.03	0.068 ± 0.01
LESG	5	0.002	0.337	0.357	0.292	980 ± 114	172 ± 36.1	0.057 ± 0.03	0.059 ± 0.01
SESG	5	0.002	0.332	0.354	0.292	894 ± 90.4	168 ± 40.2	0.063 ± 0.04	0.058 ± 0.01
WGG	5	0.002	0.349	0.366	0.294	898 ± 137	147 ± 24.3	0.046 ± 0.03	0.050 ± 0.01
Mean	57	0.002	0.347	0.371	0.303	933 ± 90.3	174 ± 36.3	0.064 ± 0.04	0.059 ± 0.01
Boran	8	0.002	0.300	0.307	0.242	826 ± 125	130 ± 22.3	0.022 ± 0.03	0.044 ± 0.01
Moroccan	10	0.002	0.257	0.277	0.233	985 ± 474	205 ± 146	0.074 ± 0.08	0.070 ± 0.05
Mean	18	0.002	0.279	0.292	0.238	905 ± 299	167 ± 84.0	0.048 ± 0.06	0.057 ± 0.03
Bengal	7	0.002	0.292	0.320	0.261	729 ± 90.3	114 ± 17.1	0.088 ± 0.06	0.039 ± 0.01
Pakistan	10	0.002	0.226	0.299	0.259	1663 ± 811	410 ± 233	0.243 ± 0.11	0.140 ± 0.08
Iranian	10	0.002	0.233	0.260	0.228	641 ± 481	186 ± 203	0.102 ± 0.08	0.064 ± 0.07
Mean	27	0.002	0.250	0.293	0.249	1011 ± 461	237 ± 151	0.144 ± 0.08	0.081 ± 0.05
Italian	10	0.002	0.216	0.286	0.257	753 ± 339	144 ± 87.5	0.246 ± 0.16	0.05 ± 0.03
Bezoar	10	0.002	0.217	0.286	0.238	1608 ± 69.0	388 ± 133	0.242 ± 0.16	0.133 ± 0.04

**Figure 1:**
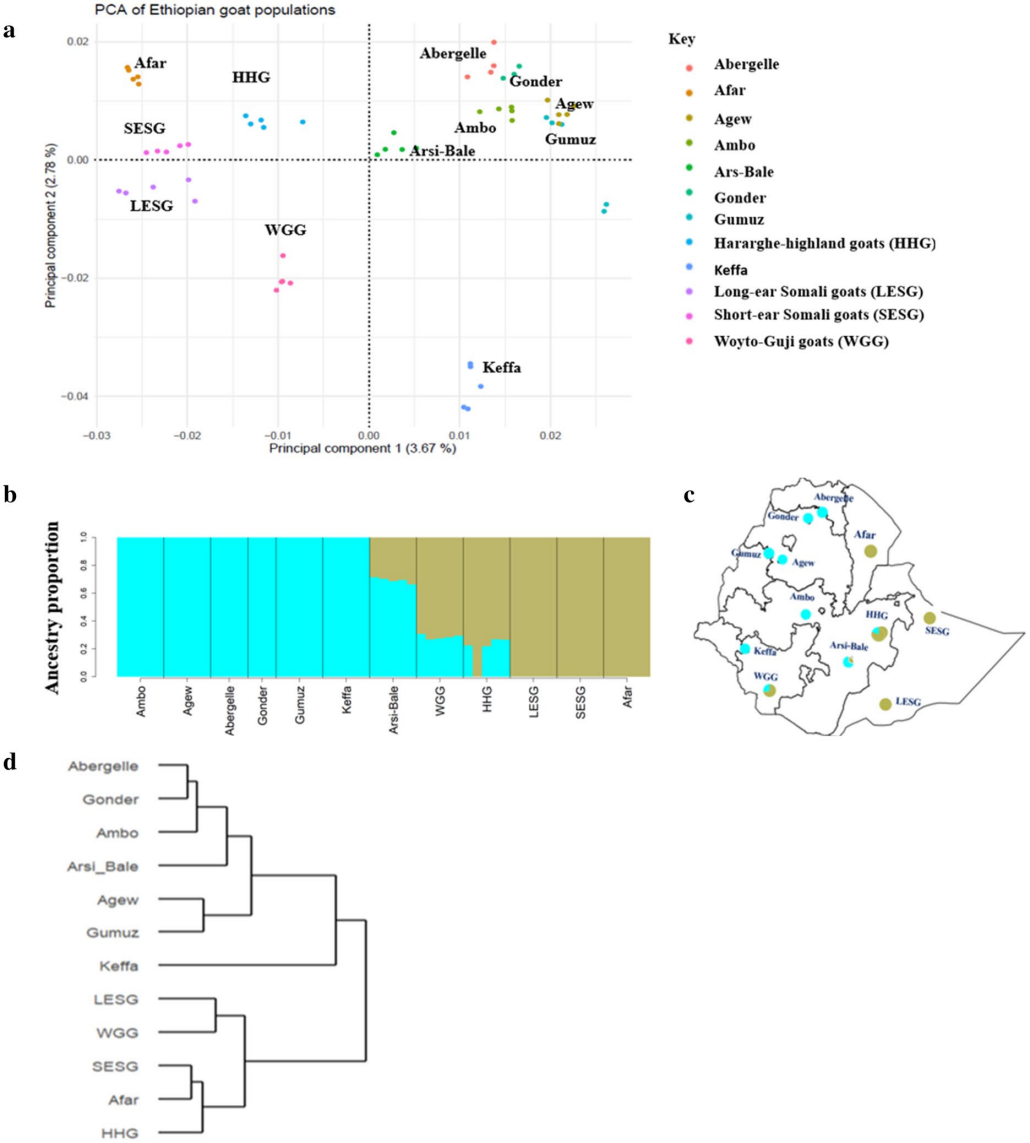

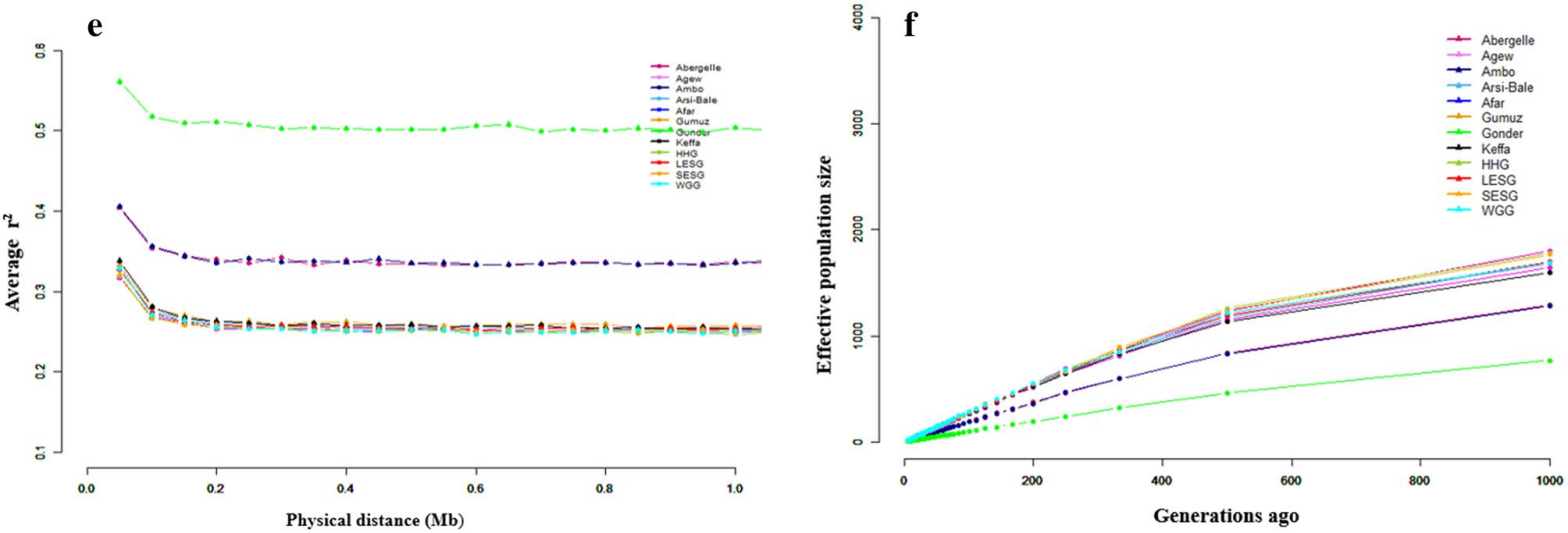
Population genetic structure and relationship of the Ethiopian goat populations based on (**a**) PCA and (**b**) ADMIXTURE analysis at K = 2, (**c**) geographic distribution and genetic admixture proportion of the Ethiopian indigenous goat populations (**d**) phylogenetic tree constructed using F_ST_ values, (**e**) the pattern of linkage disequilibrium (r^2^) from 0 to 1 Mb and (**f)** the pattern of effective population size (N_e_) in the past 1000 generations.

Π = Nucleotide diversity, H_O_ = Observed heterozygosity, H_E_ = Expected heterozygosity, D_ST_ = Genetic distance, F_HOM_ and F_ROH_ = Genomic inbreeding coefficients, ROH length = length of Run of homozygosity (RoH), SD = Standard deviation, Mb = Mega base,

Genetic structure and relationship were assessed at the first instance for Ethiopian indigenous goats only and then for the overall dataset that included all Ethiopian, non-Ethiopian and the wild Bezoar goats. PC1 and PC2 of the PCA explained 3.67% and 2.78% of the total genetic variation of Ethiopian goats (Fig. 1a) and each revealed two broad genetic clusters. The first cluster identified by PC1 includes Afar, Hararghe Highland, Short-eared Somali, Long-eared Somali, and Woyto-Guji. The second cluster of PC1 comprises Abergelle, Gonder, Agew, Ambo, Gumuz, Arsi-Bale and Keffa. Similarly, PC2 grouped together Afar, Hararghe highland, Short-eared Somali, Abergelle, Gonder, Agew, Ambo, Gumuz, and Arsi-Bale in its first cluster while its second cluster grouped together Long-eared Somali, Woyto-Guji, and Keffa. In combination, these two PC’s reveal four clusters that likely reflect a fine-scale genetic divergence in Ethiopian indigenous goats. The first one is made up of Keffa; the second comprises Long-eared Somali and Woyto-Guji; the third comprises Afar, Hararghe Highland and Short-eared Somali, and the fourth includes Abergelle, Gonder, Agew, Ambo, Gumuz and Arsi-Bale.

To further examine the genetic structure of Ethiopian indigenous goats, we generated an NJ phylogeny using F_ST_ genetic distances (Fig. 1d). This revealed two broad genetic clusters that support the PCA results. We named these two genetic clusters A and B. The F_ST_ phylogeny also supports the fine-scale and deep genetic structure in Ethiopian goats revealed by PCA with Keffa being genetically distinct. ADMIXTURE analysis showed the lowest CV error at K = 1 suggesting genetic homogeneity among Ethiopian goats. However, the genetic profile at K = 2 (Fig. 1b) shows two broad genetic clusters, whose population composition corresponds to the ones identified by PC1 of the PCA and the F_ST_ phylogeny. For brevity, we also name these A and B. At the same K value (i.e., K = 2), ADMIXTURE analysis provides additional insights into the genetic structure of Ethiopian goats that are not apparent in the PCA and F_ST_ phylogeny; that the genomes of Arsi-Bale, Woyto-Guji and Hararghe Highland comprise different proportions of clusters A and B. Cluster A predominates in Arsi-Bale (69.0%) and, B in Woyto-Guji (71.47%) and Hararghe Highland (80.47%) (Supplementary Fig. S5). An analysis of the geographic distribution of the two genetic clusters across Ethiopia shows that A predominates in the North and West, while B occurs at a higher frequency in the South and East of the country (Fig. 1c).

**Figure 2:**
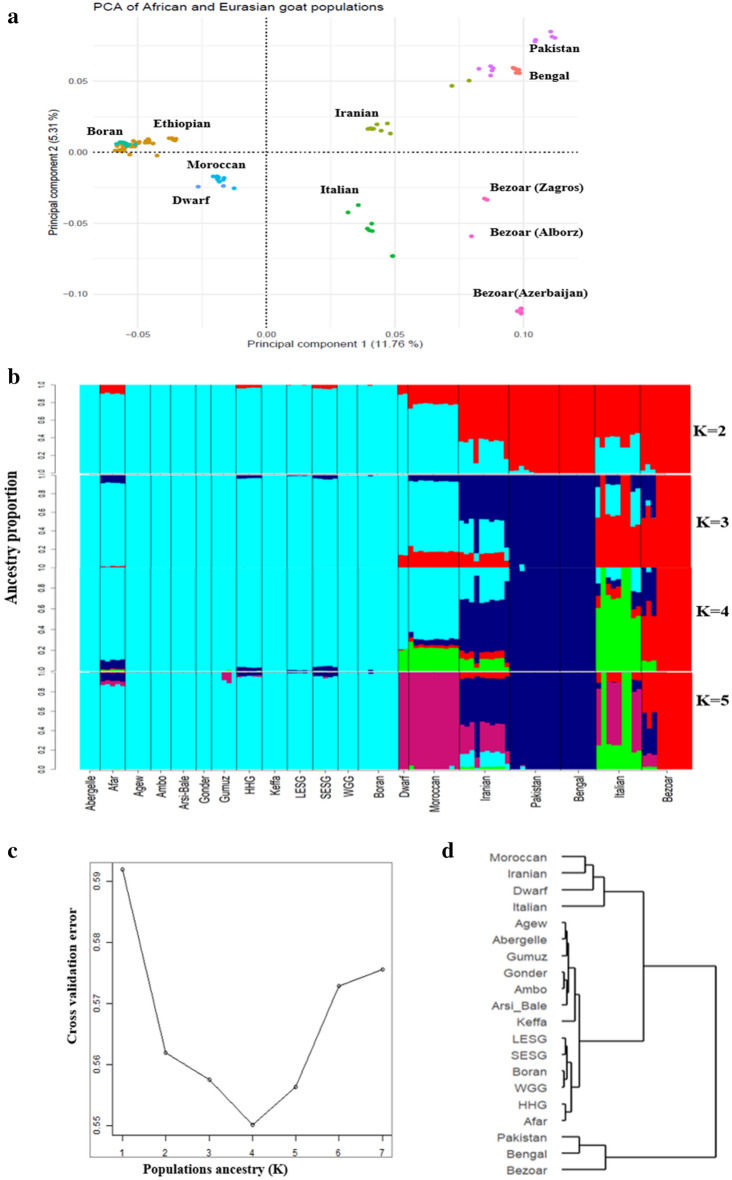

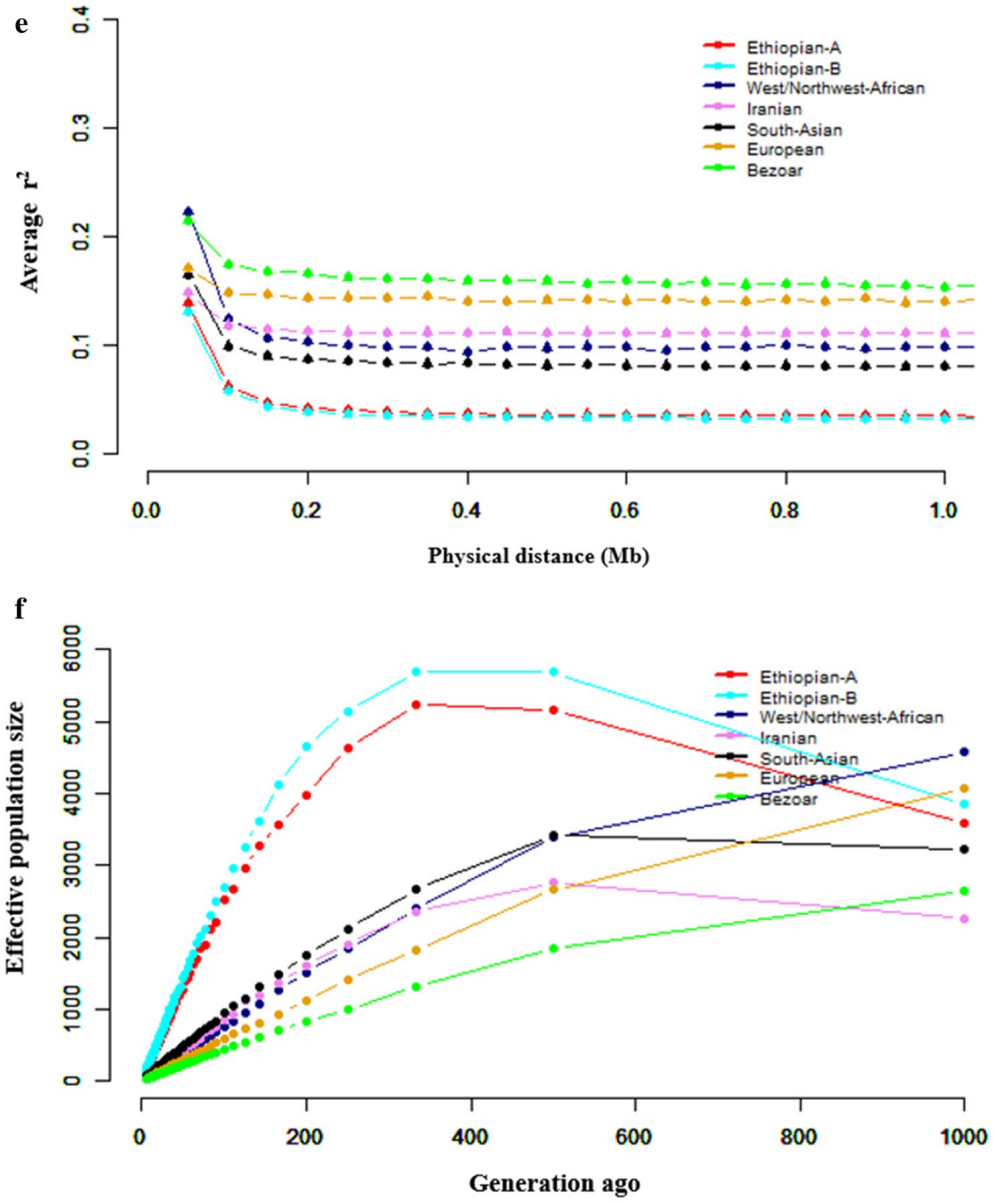
Population genetic structure and relationship of the African, South Asian, Middle Eastern, European, and wild Bezoar goat populations. (**a**) PCA, (**b**) ADMIXTURE analysis at K = 2, 3, 4, and 5, (**c**) Cross-validation error (CV) value at K = 4, (**d**) phylogenetic tree constructed using FST values, (e_a_ and e_b_) the pattern of linkage disequilibrium (r^2^) from 0 to 1 Mb and (f_a_ and f_b_) the pattern of effective population size (N_e_) in the past 1000 generations. Note: The LD and Ne are plotted based on the admixture, PCA and phylogenetic tree results. Ethiopian-A includes Abergelle, Gonder, Agew, Ambo, Gumuz, Arsi-Bale and Keffa whereas Ethiopian-B consists of Afar, Short ear Somali, Long ear Somali, Haraghe highland, Woyto-Guji and Kenyan-Boarn goats.

To explore genetic variation in Ethiopian goats in the context of their divergence from other African and non-African goat populations, we generated a PCA and ADMIXTURE profiles while including east African, west African, north-west African, European, South Asian, Middle East, and wild Bezoar goats in the analysis. PC1 and PC2 explain, respectively 11.76 and 5.31% of the total genetic variation in the dataset (Fig. 2a). It shows that Boran (Kenya) and Ethiopian goats are monophyletic. PC1 separates African and non-African goats while PC2 separates east African (Kenya, Ethiopia) goats from the west and north-west African (Dwarf and Moroccan) ones. This genetic clustering pattern is replicated by ADMIXTURE (Fig. 2b), which also reveals deeper insights into the genome architecture of the study populations. It reveals the lowest CV score is K = 4 (Fig. 2c), suggesting four genetic groups. East African goats share one genetic group that also occurs in the west (78.8%; Dwarf) and northwest (68.7%; Moroccan) African goats (Supplementary Fig. S6d). We refer to this as the “African genetic group”. The next dominant genetic group occurs in Pakistan and Bangladesh goats (referred to as the “South Asian genetic group”). The third predominates in Italian goats (“European genetic group”) whilst the fourth predominates in the wild Bezoar goat (“Wild genetic group”). We observed a certain level of admixture of the four genetic groups in Iranian goats. Admixture is also observed in west and north-west African, and European goats, and in a few individuals of the wild Bezoar goats (Fig. 2b; Supplementary Fig. S5d). Further scrutiny of ADMIXTURE at K = 5 shows that west and north-west African goats share a genome component that is different from that of their east African counterparts (Fig. 2b). This suggests further divergence of the African genetic group into two sub-groups, an east African, and a west/north-west African one. The latter is also found in Iranian and European goats and the wild Bezoar goats but at low frequencies.

### Genome-wide dynamics

Genome-wide demographic dynamics were inferred by assessing LD patterns against genomic distances and changes in N_e_ over generation time for each population and genetic clusters that were revealed by PCA and ADMIXTURE. The pattern of LD decay was the same for all the population clusters (Figs. 1e, 2e). It reveals higher LD at shorter genomic distances which decays rapidly reaching a plateau around ~ 0.2 Mb. Generally, Ethiopian goats show higher average LD (r^2^ ≥ 0.25) (Fig. 1e) and within them, Gonder had the highest LD (r^2^ ≥ 0.5), followed by Ambo and Abergelle (r^2^ ≥ 0.34) and then the other populations (r^2^ ≥ 0.25).

The pattern of N_e_ was similar across all the Ethiopian indigenous goat populations (Fig. 1f). There was a gradual decline in N_e_ between 1000 and 500 generations ago, following which the decline accelerated. Performing the analysis based on the genetic groups generated by cluster analysis reveals that both clusters A and B of Ethiopian populations show an increase in N_e_ between 1000 and 400 generations ago following which there is a drastic decline (Fig. 2f).

### Selection signature analysis

**Figure 3:**
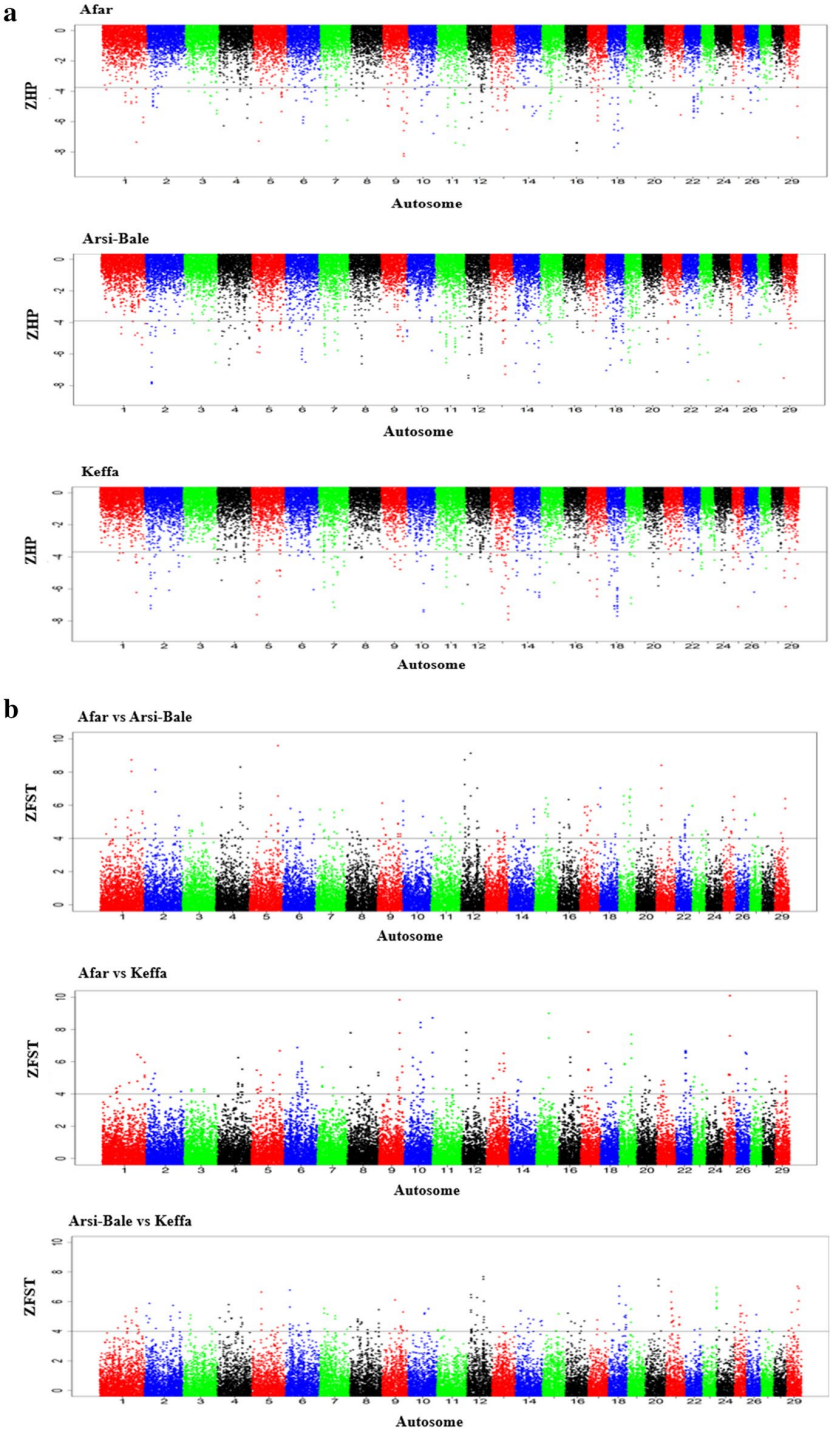

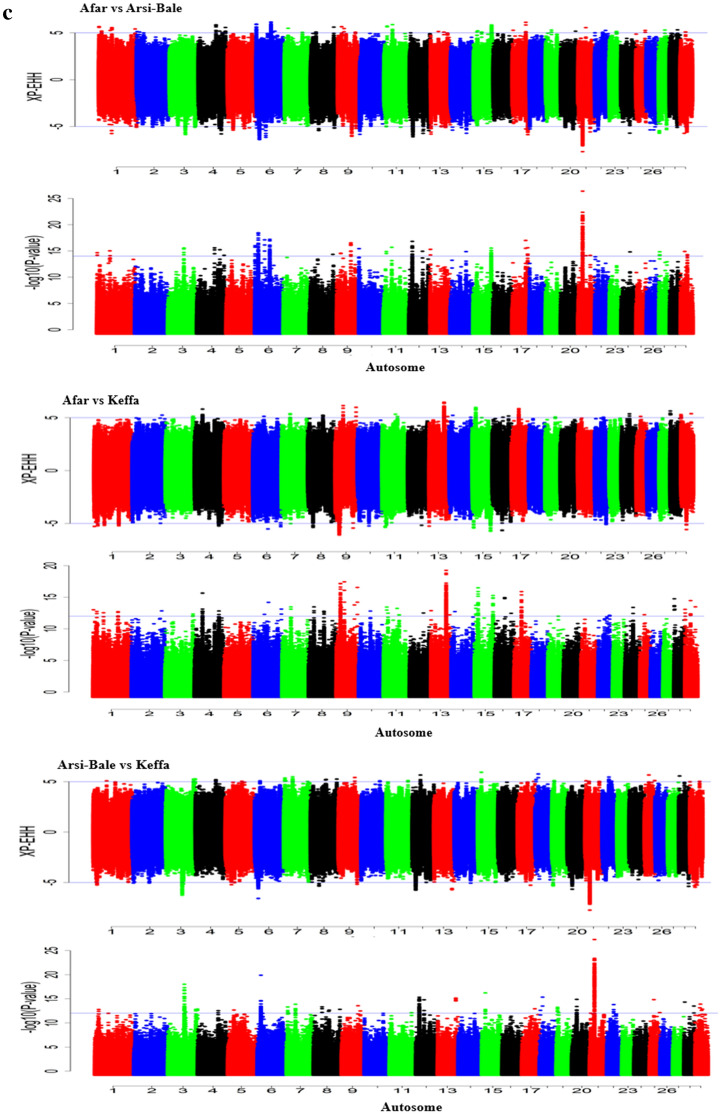
Manhattan plots showing genome-wide selection signals as revealed by: (**a**) ZHP, (**b**) ZF_ST_ and (**c**) XP-EHH amongst Ethiopian indigenous goat populations. (**a**) ZHp Analysis for individual Ethiopian goat populations (Afar, Arsi-Bale and Keffa). (**b**) Manhattan plots for pairwise ZF_ST_ analysis results among the three Ethiopian indigenous goat populations (Afar, Arsi-Bale, Keffa) used in this analysis. (**c**) Manhattan plots for pairwise XP-EHH analysis results among the three Ethiopian indigenous goat populations (Afar, Arsi-Bale, Keffa) used in this analysis.

We investigated genome-wide selection signatures with Hp, F_ST,_ and XP-EHH tests to explore whether the population clusters observed in Ethiopian goats are the result of adaptation to different environments. For this analysis, we selected Afar, Arsi-Bale, and Keffa goats as they occurred in different clusters on the PCA. The H_P_ test revealed a total of 196 candidate regions under selection, that overlapped 484 genes across Afar, Arsi-Bale, and Keffa goats (Fig. 3a; Supplementary Table S5). The F_ST_ (Fig. 3b; Supplementary Table S6) and XP-EHH (Fig. 3c; Supplementary Table S7) tests identified a total of 222 and 356 candidate regions, respectively that spanned 411 and 757 genes when contrasting Afar and Arsi-Bale, Afar and Keffa, and Arsi-Bale and Keffa. Based on the ARS1 RefSeq gene annotation, a total of 145 regions did not overlap with any gene(s) and/or spanned genes that are not yet annotated (Supplementary Tables S5–S7). Given the large number of candidate regions identified by the three tests, to retain high specificity we used the threshold score values of − 7.0 (ZH_P_), 7.0 (ZF_ST_), and 6.0 (XP-EHH) to define the top-most significant selection regions. We regarded these to be the primary selection signatures that are shaping the genomes of the study populations. Our results and discussions will be based on these top-most significant regions unless specified otherwise.

**Figure 4:**
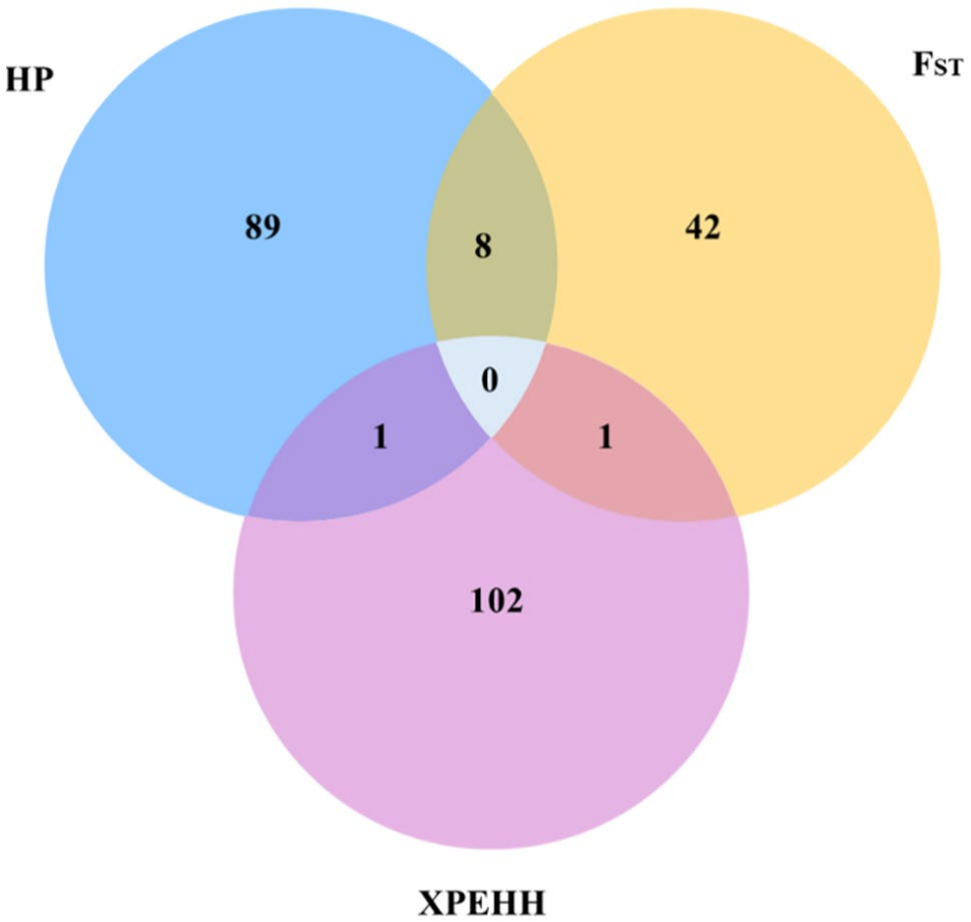
Venn diagram showing the number of genome-wide selection signals in Ethiopian indigenous goats that are specific to, and shared between H_P_, F_ST,_ and XP-EHH approaches.

Based on the above cut-off threshold scores, eight candidate regions in Afar (overlapping 36 genes), seven in Arsi-Bale (seven genes), and eight in Keffa (55 genes) were identified by ZH_P_ as the top-most significant regions (Table 2). The ZF_ST_ identified nine regions in Afar vs Arsi-Bale (13 genes), nine in Afar vs Keffa (34 genes), and four in Arsi-Bale vs Keffa (4 genes) (Table 3) while those identified by XP-EHH were, 23 for Afar vs Arsi-Bale (80 genes), seven for Afar vs Keffa (12 genes), and nine for Arsi-Bale vs Keffa (12 genes) (Table 4). Of these 253 genes, eight (*SCNNIB, SMPD3, NUP43, ZNF609, COG7, ZFP90, PCMT1, PIFI*) overlapped between ZH_P_ and ZF_ST,_ one each (Fig. 4) overlapped between ZH_P_ and XP-EHH (*BPIFB4),* and ZF_ST_ and XP-EHH (*ALOX5AP*).

**Table 2:** Candidate regions revealed to be under selection and associated genes as identified by the ZHp approach in Afar, Arsi-Bale and Keffa Ethiopian indigenous goat populations.

Chromosome	Region (Mb)	Highest ZHp score	No. of candidate regions	Genes
Afar				
1	119.60–119.80	− 7.3551	3	
7	21.60–21.90	− 7.2470	5	
9	73.85–74.10	− 8.2640	4	*NUP43*, *PCMT1*
11	64.90–65.10	− 7.3977	3	*TRNAC-ACA*
16	40.90–41.15	− 7.9183	4	*MTOR*, *ANGPTL7*, *EXOSC10*, *SRM*, *MASP2*, *TARDBP*
18	22.95–23.15	− 7.6813	3	*CHD9*
18	36.45–36.80	− 7.4372	6	*CTCF*, *CARMIL2*, *ACD*, *PARD6A*, *ENKD1*, *GFOD2*, *RANBP10*, *TSNAXIP1*, *CENPT*, *THAP11*, *NUTF2*, *EDC4*, *NRN1L*, *PSKH1*, *PSMB10*, *LCAT*, *SLC12A4*, *DPEP3*, *DPEP2*, *DDX28*, *DUS2*, *NFATC3*
29	46.60–46.70	− 7.0457	1	*ALDH3B1*, *NDUFS8*, *TCIRG1*, *CHKA*
Arsi-Bale				
2	21.15–21.50	− 7.8828	5	
12	10.05–10.25	− 7.5186	3	GPR183, GPR18, UBAC2
14	73.60–73.75	− 7.0998	2	SLA, TG
	90.20–90.40	− 7.8080	3	LMBRD1
18	2.00–2.15	− 7.0364	2	
20	51.70–51.95	− 7.1316	4	
23	30.45–30.60	− 7.6424	2	SUPT3H
Keffa				
2	20.95–21.30	− 7.2204	6	
5	18.05–18.60	− 7.6087	2	KITLG
10	57.55–57.75	− 7.4240	3	OAZ2, ZNF609
13	61.70–61.95	− 7.9138	4	BPIFB4
18	26.10–27.00	− 7.2406	6	ADGRG5, CCDC102A, SLC12A3, NUP93, ADGRG1, HERPUD1, ADGRG3
	36.00–37.20	− 7.6909	18	*CBFB, C16orf70, B3GNT9, TRADD, HSF4, NOL3, KIAA0895L, EXOC3L1, E2F4, ELMO3, MIR328, TMEM208, FHOD1, SLC9A5, PLEKHG4, KCTD19, LRRC36, TPPP3, ZDHHC1, RANBP10, TSNAXIP1, CENPT, THAP11, NUTF2, EDC4, NRN1L, PSKH1, PSMB10, LCAT, SLC12A4, DPEP3, DPEP2, DDX28, DUS2, NFATC3, ESRP2, PLA2G15, SLC7A6, SLC7A6OS, PRMT7, SMPD3, ZFP90*
25	20.90–21.10	− 7.1041	3	*SCNN1B, COG7*
29	5.00–5.15	− 7.1025	2

**Table 3:** Candidate regions under selection and associated genes as identified by the ZF_ST_ approach in Afar x Arsi-Bale, Afar x Keffa and Arsi-Bale x Keffa Ethiopian indigenous goat populations.

Chromosome	Region (Mb)	Highest ZF_ST_ score	No. of candidate regions	Genes
Afar x Arsi-Bale				
1	111.20–111.45	8.7369	4	*GMPS, SLC33A1*
2	39.75–40.00	8.1483	4	*PLEKHM3, FZD5, CREB1*
4	87.45–87.90	8.3029	7	*ABCB4, RUNDC3B*
5	101.50–102.10	9.5972	6	*FGF23, TIGAR, CCND2*
12	13.65–13.95	8.7414	5	
	35.15–35.30	9.1363	2	
	57.25–58.35	7.0350	3	*ALOX5AP, N4BP2L2, PDS5B*
18	2.00–2.15	7.0405	2	
21	19.00–19.20	8.4123	3	
Afar x Keffa				
8	10.70–10.85	7.7960	2	*SCARA5, ESCO2*
9	73.85–74.35	10.7976	5	*LATS1, NUP43, PCMT1*
10	57.45–57.75	8.4319	5	*ZNF609, OAZ2, PIF1*
	100.80–101.00	8.7218	3	
12	13.70–13.95	7.8119	3	
15	45.95–46.15	8.9992	3	*SOX6*
17	26.00–26.65	7.8414	4	*ULK1, PUS1, EP400, SNORA49, FBRSL1, LRCOL1, P2RX2*
19	42.25–42.65	7.6954	4	*NAGLU, HSD17B1, COASY, MLX, PSMC3IP, RETREG3, TUBG1, CNTNAP1, EZH1, RAMP2, VPS25, WNK4, CNTD1, PSME3, AOC2, G6PC1*
25	20.85–21.10	10.4511	4	*SCNN1B, COG7*
Arsi-Bale x Keffa				
12	57.20–57.40	7.6788	3	
18	37.00–37.25	7.0389	4	*SMPD3, ZFP90, CDH3, SA*
20	45.55–45.75	7.4978	3	
29	38.20–38.35	7.0217	2

**Table 4:** Candidate regions under selection and associated genes as identified by the XP-EHH approach in Afar x Arsi-Bale, Afar x Keffa and Arsi-Bale x Keffa Ethiopian indigenous goat populations.

Chromosome	Region (Mb)	Highest XP-EHH Score	No. of candidate regions	Genes
Afar x Arsi-Bale				
1	55.00–55.15	6.3462	1	*NECTIN3*
	155.9–157.05	6.1671	2	*EFHB, RAB5A, PP2D1*
3	65.95–66.60	6.0621	2	
6	40.60–40.75	6.9709	1	*PACRGL*
9	58.50–58.70	6.5111	1	*EYA4*
11	12.85–13.00	6.5781	1	*DYSF*
	87.10–87.25	6.0839	1	*CYS1, KLF11*
12	13.20–14.10	6.1591	2	*DNAJC3, DZIP1, CLDN10*
	56.10–57.75	7.0985	3	*SNORA70, KATNAL1, USPL1, ALOX5AP, RXFP2*
13	32.25–32.40	6.5657	1	*CACNB2*
15	15.25–18.00	6.7408	4	*RAG2, RAG1, TRAF6, LDLRAD3, ELF5, CAT*
17	3.65–3.80	6.8765	1	*TTC28*
19	24.10–24.25	6.4008	1	*TRPV1, SHPK, CTNS, TAX1BP3, EMC6, P2RX5, ITGAE, HASPIN*
20	61.95–62.10	6.6412	1	*CTNND2*
21	11.60–12.45	6.2535	2	
	18.90–19.30	7.8276	1	*ISG20*
22	2.55–2.70	6.6094	1	*CMC1, AZI2*
23	32.85–33.35	6.1298	2	*MRPS10, GUCA1B, GUCA1A, GUCA1ANB, C6orf132, TAF8, FOXP4*
25	38.90–39.05	6.5969	1	*AIMP2, EIF2AK1, ANKRD61, USP42, CYTH3*
26	31.10–33.15	6.2570	2	*NKX23, GOT1, CNNM1, CRTAC1, RRP12, FRAT2, FRAT1, ARHGAP19*
29	17.35–19.40	6.1719	3	*ALG8, NDUFC2, THRSP, KCTD14, INTS4*
	21.25–24.05	6.1197	4	*PRMT3, HTATIP2*
	26.10–28.60	6.1250	3	*VWA5A, NRGN, VSIG2, ESAM, MSANTD2, ROBO3, ROBO4, STT3A, CHEK1, ACRV1, SOLD1, PATE1, PATE2, PATE3, PUS3, HYLS1*
Afar x Keffa				
1	1.70–2.15	6.9002	2	*HUNK*
2	26.85–27.20	6.0046	2	
	114.80–116.35	6.1629	2	
9	73.95–74.85	6.7211	2	*PPP1R14C, IYD*
11	12.85–13.00	6.5923	1	*DYSF*
13	61.60–62.00	6.5096	1	*SUN5, BPIFB2, BPIFB6, BPIFB3, BPIFB4*
15	5.60–5.75	6.3199	1	*NDUFS3, KBTBD4, PTPMT1*
Arsi-Bale x Keffa				
3	118.65–118.80	6.1374	1	*POU2F1*
8	5.30–5.65	6.1761	2	*GALNT7*
12	54.95–58.90	6.2176	5	*USPL1, ALOX5AP, RXFP2, FRY*
15	13.45–14.70	6.0397	2	
	70.85–72.6	6.2050	2	
18	3.20–3.35	6.4696	1	*GLG1, RFWD3*
19	4.95–5.10	6.4695	1	*STXBP4*
27	43.30–43.35	6.0564	1	*CLN8*
28	2.95–3.10	7.5618	1	*MAPK8, FRMPD2*

The 253 genes were compared with published literature to determine their functional significance. We categorised these genes into two groups (Tables 2, 3 and 4). Group 1 comprises genes previously reported in other livestock species: among these are five genes: *POU2F1* and *KITLG*, which relate to coat/fur/skin pigmentation, *RXFP2* which is a major gene for sheep horn status and secondary sexual characteristics, *CACNB2* which plays a role in reproduction process in dairy cows, *PPP1R14C* which is believed to contribute to trypanotolerance in Sheko cattle. Group 2 consisted of genes reported to have biological roles in other animal species. These included among others thermo-sensitivity (*TRPV1*, *PCMT1*, *IYD*, *PACRGL*), oxidative stress response and control of reactive oxygen species (*PCMT1*, *TIGAR*, *TRPM2*, *ALDH3B1*, *ALOX5AP*, *RAMP2*, *HSF4*), hypoxic survival and adaptation to high-altitude (*DDX28*, *RUNDC3B*, *PIK3CD*, *TIGAR*, *PTPMT1*, *STXBP4*), and reproduction function-related (*NUP43*, *EXOSC10*, *TARDBP*, *DPEP3*, *ESCO2*, *OAZ2*, *HSD17B1*, *PSMC3IP*, *EZH1*, *NECTIN3*, *KATNAL1*, *CACNB2*, *HASPIN*, *USP42*, *SUN5*, *KCTD19*, *ELF5*, *KITLG*, *TSNAXIP1*) genes. Generally, the majority of these genes in both classes encode proteins with multifaceted functions that range from adaptation, development, regulation, and maintenance of tissue and cellular functions.

Functional annotation and gene ontology analysis were performed with DAVID at two levels (1) for all the genes identified by ZH_P_ in each population and (2) for all the genes identified by the three comparative analyses (Afar vs Arsi-Bale, Afar vs Keffa, and Arsi-Bale vs Keffa) involving ZF_ST_ and XP-EHH combined as one gene list. We found several functional gene clusters that were significantly enriched (Supplementary Tables S8 and S9). In Afar, the enriched clusters were RNA degradation, endocytosis, autophagy, Rap1 signaling pathway, PI3K-Akt signaling pathway, and MAPK signaling pathways. In Arsi-Bale, enrichments were Ras signaling pathway, Rap1 signaling pathway, PI3K-Akt signaling pathway, MAPK signaling pathway, bacterial invasion of epithelial cells and melanogenesis. In Keffa, MAPK signaling pathway, glycerophospholipid metabolism,TGF-beta signaling pathway, Yersinia infection and IL-17 signaling pathway were overrepresented. In the Afar vs Arsi-Bale comparative analysis, significantly enriched clusters were for neurological system processes, apoptotic processes involved in morphogenesis, and sensory organ development. In Afar vs Keffa, the significantly enriched clusters were for sensory organ development, system process, and cell differentiation. Finally, Arsi-Bale vs Keffa showed distinct and extensive enrichments for 104 clusters, including growth, hematopoietic or lymphoid organ development, tissue development, and multicellular organismal homeostasis.

## Discussion

Following goat domestication from the wild Bezoar ~ 11,000 years ago^1,2^, population bottlenecks, inbreeding, intermixing, and selection (natural and artificial) have been modifying the genomes of domestic goats. This is especially the case in Africa where their initial introduction as exotics from the centre of domestication into the continent exposed them to novel environments and physiological extremes and challenges. Here, by analysing genetic divergence by exploiting SNPs from 57 individual genomes from 12 Ethiopian indigenous domestic goat populations and placing them within a comparative dataset of published African and non-African domestic goat breeds and the wild Bezoar goat allowed us to contextualize regional, continental, and intercontinental diversity and divergence. Notwithstanding differences in sequencing depth and platforms, our sequence statistics (Supplementary Table S1) were consistent with previous observations in goats^18–20^, sheep^21,22^, and cattle^23,24^, indicating the high quality and reliability of our dataset.

Irrespective of the species and markers used, several studies have reported high genetic diversity estimated from whole-genome sequences in indigenous livestock compared to exotic/commercial breeds^22,25,26^. The reduced genetic diversity in the latter is the outcome of long-term artificial selection and/or genetic drift due to a demographic history of low effective population sizes. The genome-wide autosomal SNPs showed high values in all the estimated parameters of genetic diversity in Ethiopian goats (Table 3). These values are comparable with those reported for indigenous goats in Uganda^27^, South Africa^28^, Egypt^14^, Spain^29^ and Italy^30^ estimated using SNP microarray genotypes and from whole-genome sequence analysis in indigenous African goats^25^, cattle^24^ and sheep^22^. In diploid genomes, RoH represents continuous homozygous segments of DNA sequences and can provide insights into how population history, structure, and demography have evolved over time^31^. In this study, estimates of F_RoH_ and F_HOM_ reveal low levels of inbreeding in all the populations analysed which is consistent with findings in Ugandan^32^ and Egyptian Barki goats^14^. Our analysis also revealed a similar pattern of high frequency (> 50%) of the shortest average RoH length segments (0.1–0.25 Mb length category) in the populations analysed (Supplementary Table S4; Supplementary Figs. S3, S4). This skewed distribution agrees with other findings on goats^32,33^, sheep^34^ and cattle^34–36^ where long RoH segments were more infrequent than shorter ones. Short ROHs suggest inbreeding is not recent. Taken together, these results appear to suggest that the high heterogeneity in the study populations could be the result of a combination of factors. These include low inbreeding due to random mating and historic admixture arising from the communal use of resources and/or the common practice of sharing and/or gifting stock to cement social bonds and relationships, and the predominance of natural selection which favours standing genetic variation. This is supported by the demographic dynamics, which show all populations have low LD ranges (> 200 kb) and historic high N_e_ (Fig. 2). The highest average LD was recorded in Ethiopian (0.25–0.55) goat populations. These LD values are within the range reported for a large number of goat breeds^37^ and East African shorthorn Zebu cattle^38^. However, when the data is combined the lowest and highest LD values are recorded in Ethiopian and wild Bezoar goats, respectively. The low levels of long-range LD show the lack of intensive selection or the populations have had large effective ancestral population sizes^39^. The decline in N_e_ from around 1250 years ago (500 generations) is difficult to explain as no significant events that could have affected goat populations have been reported in the Horn of Africa region. We however speculate that the start of this decline could have been driven by the Lapanarat–Mahlatule drought that occurred in the region and was characterized by a sequel of severe droughts and political upheavals^40^.

Though not native to Africa, goats are ubiquitous in the continent and are closely associated with the subsistence practices, socio-cultural life, and economy of many African societies. Archaeological evidence suggests that goats first arrived in Africa from Southwest Asia^41^. Our phylogenetic and population structure analyses at the whole genome level revealed at least two ancestral genetic groups in African goats; one was observed in west and north-west African goats and the other in east African goats. We hypothesise that these two genetic ancestries could be contiguous with the trajectories of the initial movement of the first goat-farming pastoralists into the continent from Southwest Asia. Radiocarbon dates of caprovid remains from the North African Mediterranean coastline are amongst the oldest in the continent^6,42^. Thus, the west/north-west African ancestry, due to its location furthest from the postulated initial entry point of goats into Africa, was likely the first in the continent. To arrive at their current locations this ancestral group may have dispersed along two routes following its entry via Egypt. The ancestry that occurs in north-west African goats dispersed along Africa side of the Mediterranean Sea while the one found in west Africa dispersed overland across the present-day Sudano-Sahelian belt^43^.

The East African genetic ancestry could represent goats that spread from the Near East into the central Sahara, Sudan Nile, and the Ethiopian highlands between 6500 and 5000 BP^6,7^. This genome ancestry could have spread overland via the Sinai Peninsula and Nile Delta regions and/or via the Red Sea Hills region of the Egyptian Red Sea coast. The split of Ethiopian goats into two clusters, named here A and B (Fig. 1a,b,d), and their respective geographic distribution across the country could shed light on the dispersal of this genomic ancestry into East Africa. The two clusters mirror findings from the analysis of 50 K SNP genotype data of Ethiopian goats^15^ and of mitochondrial DNA, which identified two haplogroups in the Ethiopian^11^, Kenyan^10^, Sudanese^44^ and Egyptian^45^ indigenous goats. Cluster A predominates in goats found in the north and west of Ethiopia, while B occurs at a higher frequency in goats found in the south and east of the country (Fig. 1d). Their spread across the country was most likely facilitated by socio-cultural and commercial interactions as can be inferred from anthropologic, linguistic and human genetic studies^46^. This geographic spread led us to suggest that cluster A could have dispersed to Ethiopia from Egypt following the Nile River basin or across the Red Sea Hills, while cluster B most likely arrived in Ethiopia via the Horn of Africa through the Bab el-Mandeb strait.

Worth mentioning is that the inclusion of genomes from east, west, and north-west Africa, Europe, South Asia, the Middle East, and from the wild Bezoar goats provided an interesting insight. The ADMIXTURE analysis showed that all the four genomes it revealed are present in Iranian (Middle East) goats, which supports this as the cradle of present-day goat genome diversity.

Ethiopia is characterized by a diverse combination of agro-eco-climates and ancient and modern human ethnic diversity that may have influenced the genome architecture of indigenous livestock. Our phylogenetic analysis revealed fine-scale genetic structuring in Ethiopian goats, which we hypothesize could be driven by environmental adaptation. Thus, signatures of selection were investigated within and between Afar, Arsi-Bale, and Keffa on the premise that their divergence is driven by adaptation to contrasting environments and therefore can serve as good proxies for investigating selection signatures resulting from genomic divergence. Afar goats inhabit a low altitude area (120–200 masl) with a hot semi-arid/arid agro-ecology (mean annual rainfall 150–300 mm). Arsi-Bale goats inhabit the Bale mountains (> 3000 masl) with a cool, cold sub-humid and alpine agro-ecology. These two populations clustered separately in the PCA suggesting genomic divergence. Keffa, which also occurs in a mid-altitude (≤ 1800 masl) environment, showed a clear genetic divergence from the other Ethiopian populations, suggesting it is genetically unique. This was observed previously by Tarekegn^11^ from the analysis of 50 K SNP genotype data. The selection signature analysis revealed several candidate genomic regions under selection, some of which did not span any genes. This is not uncommon; it has been reported in cattle^47^, sheep^14^, and goats^15^. In line with our hypothesis, the top-most candidate regions under selection, spanned genes with roles in adaptation to different biophysical stressors rather than production. This suggests that natural rather than deliberate artificial selection is the principal driver of divergence in the studied populations and is mediated by the concurrent action of a complex network of genes. The large number of candidate regions and genes detected is also not surprising. Similar results have been reported for livestock species from extreme environments^21,48^.

Our findings corroborate those of Tarekegn et al.^15^ who reported the divergence of Keffa from other Ethiopian goats by analysing 50 K SNP genotype data suggesting it to be genetically distinct. The authors speculated that it was due to Keffa being trypanotolerant. Whereas, we found a large number of selection signals in Keffa, Tarekegn et al*.* found none. We attribute this difference to the higher resolution afforded by whole-genome sequences. The XP-EHH between Afar vs Keffa detected a strong selection region on CHI9 (73.95–74.85 Mb) spanning two genes, *PPP1R14C* and *IYD*. Interestingly, *PPP1R14C* was found in a selection sweep region in the Ethiopian Sheko cattle and it was postulated to be one of the candidate genes contributing to trypanotolerance in the breed^49^. Both Sheko cattle and Keffa goats occur in an area where trypanosomosis is an economically important livestock disease. Whether this signature presents convergent evolution in the two species for trypanotolerance is difficult to say from the current data. Cattle and goats are both bovids with minor genomic differences^50,51^ indicating minor genomic differences since their divergence from a common ancestor^14^. Therefore, it is not unusual to find genetic and biological similarities between the two species. While this signature could contribute to the uniqueness of Keffa goats, we cannot conclude it is the only factor.

Afar goats reside in a semi-arid and arid environment. It is characterized by complex interacting biophysical stressors including heat, physical exhaustion, direct solar radiation, and resource (feed and water) scarcity. It is thus unsurprising that the selection tests for Afar goats, revealed signatures that spanned genes for thermo-sensitivity e.g., *TRPV1*, *PCMT1*, *PACRGL*, and *IYD*. The activity of *PCMT1* was reported to peak under lethal temperatures, suggesting a role for the enzyme in short-term responses to heat extremes^52^. D’Alessandro et al.^53^ demonstrated that *PCMT1* also plays an essential role that ensures normal RBC circulation during oxidative stress. *TRPV1*, a thermally activated ion channel, plays a major role in thermosensation, thermoregulation, and nociception^54^, and its activation provides a high-temperature noxious-heat-avoidance signal^55,56^. We also found strong selection signatures in regions overlaying *ANGPTL7* and *ENKD1*, which are associated with maintaining skin integrity. By regulating extracellular matrix formation coupled with its high expression in keratinocytes^57^, *ANGPTL7* plays a central role in skin homeostasis, repair, and regeneration^58^. *ENKD1* regulates spindle orientation in basal keratinocytes, which promotes epidermal stratification and regeneration^59^. The epidermis protects an organism from extremes of the external environment, reduces water and heat loss and pathogen entry^60^. Thus, in arid environments, skin integrity is important for protection against solar radiation, maintaining internal homeostasis and moisture loss.

In several of the top-most candidate regions in Afar, we also found five genes, *FGF23*, *TIGAR*, *RAMP2*, *SLC12A3,* and *WNK4,* which are critical in regulating mineral and nutritional metabolism and homeostasis, and water balance. *FGF23* regulates mineral homeostasis and plays an essential physiological role in phosphate and vitamin D metabolism^61^. Bensaad et al.^62^ showed that *TIGAR* can lower intracellular reactive oxygen species in response to nutrient starvation or metabolic stress, and functions to inhibit autophagy. The adrenomedullin (AM) and its receptor-modulating protein, *RAMP2*, facilitate early adaptation to cardiovascular stress by maintaining and regulating cardiac mitochondria and cardiovascular homeostasis against cardiovascular stress ^63^. This is critical in countering physical stress resulting from long-distance trekking in search of pasture and water which can put pressure on cardiomyocytes. The *AM-RAMP2* system also suppresses endoplasmic reticulum stress-induced tubule cell death, thereby exerting a protective effect on kidneys^64^. Kidneys are critical in water and electrolyte (Na^+^, Cl^−^, and K^+^) homeostasis. *SLC12A3* encodes the thiazide-sensitive sodium chloride cotransporter, which is primarily expressed in the kidney, intestines, and bones. It plays a key role in sodium, potassium, and blood pressure regulation in response to various hormonal and non-hormonal stimuli^65^. *WNK4* functions as a molecular switch that varies the balance between NaCl reabsorption and K^+^ secretion to maintain integrated homeostasis^66^. It plays a key role in coordinating the activities of flux pathways, which are regulated by the renin–angiotensin–aldosterone system, to achieve integrated fluid and electrolyte homeostasis (osmoregulation) in the distal nephron and distal colon^67,68^ following acute dehydration and rapid rehydration.

Arsi-Bale and Keffa goats are found in Bale and Keffa zones, respectively. Keffa goats live between 1000 and 1800 masl, while Arsi-Bale goats inhabit elevations between 3000 and 4377 masl. These zones are characterized by high levels of precipitation. High-altitude environments impose a selective constraint in the form of hypobaric hypoxia^69^, which results in insufficient oxygen supply in body tissues. This would affect normal physiological functions and can result in organ failure and death^70,71^. Our selection signature analyses for Arsi-Bale and Keffa goats revealed several strong signatures that spanned genes such as *RUNDC3B*, *TIGAR*, *PTPMT1*, *STXBP4,* and *ALOX5AP* relating to hypoxia adaptation. It has been shown that *RUNDC3B* is amongst genes with variants associated with increased risk of high-altitude polycythemia (HAPC) in Tibetan dwellers^72^. HAPC is characterized by excessive proliferation of circulating erythrocytes due to high-altitude hypobaric hypoxia. The compensation for prolonged hypobaric hypoxia exposure is the main reason for the change in erythrocyte production and haemoglobin concentration that elevates oxygen retention, transportation, and exchange. Findings by Kimata et al.^73^ showed that *TIGAR* is a significant mediator of cellular energy homeostasis (glycolysis) and cell death (apoptosis) under ischemic/hypoxic stress. Through a genome-wide CRISPR-Cas9 KO library screening, Bao et al.^74^ identified *PTPMT1*, an important enzyme for cardiolipin synthesis, as the third most significant gene for hypoxic adaptation/survival, ranking right after *HIF-1α* and *HIF-1β*. In a genome-wide study of genetic adaptation to high altitude in feral Andean Horses, the highest region of allele frequency divergence spanned, amongst five other genes, *STXBP4* and *COX11*^75^. A highly significant association was also found between these two genes and are in strong LD in both humans and horses. *COX11* is up-regulated in chronic hypoxia suggesting a role in dealing with oxygen deficit, possibly by acting as a heme biosynthetic enzyme that transports copper to heme^76,77^. Its strong association and LD with *STXBP4* may suggest a similar function for this gene. *ALOX5AP* was identified in sheep as a potential candidate for climate-mediated adaptation^78^. In humans, a mutation in *ALOX5AP* was associated with lung function^79^. Given the high altitude and restricted oxygen concentration in the Ethiopian highlands, *ALOX5AP* may play a role in adaptation by modulating respiratory function.

Extreme environments (high-altitude, semi-arid, and arid) impose anatomical, physiological, and metabolic challenges with strong evolutionary pressure due to long-term exposure to acute and chronic stress and other factors dependent on the natural history of a population. These factors are an underlying constant in the three test populations (Arsi-Bale, Keffa, Afar) and what is likely driving their difference is their exposure to biotic and abiotic stress factors in contrasting environments. It is therefore insightful that some of the strongest selection signals spanned genes for oxidative stress mitigation (*TRPM2*, *DDX28*, *ALDH3B1*, *TIGAR*, *ALOX5AP*, *RAMP2*, *POU2F1*, *HSF4*), DNA damage repair and maintenance of genome stability (*EXOSC10*, *CTCF*, *FZD5*, *PIF1*, *FBRSL1*, *HSF4*), skin pigmentation and characteristics (*FBRSL1*, *EZH1*, *POU2F1*, *SOX5*, *KITLG*, *OAZ2*, *HSF4*) and reproduction function (*NUP43*, *EXOSC10*, *TARDBP*, *DPEP3*, *ESCO2*, *OAZ2*, *HSD17B1*, *PSMC3IP*, *EZH1*, *NECTIN3*, *KATNAL1*, *CACNB2*, *ELF5*, *HASPIN*, *USP42*, *SUN5*, *KITLG*, *KCTD19*, *TSNAXIP1*). Long-term exposure to acute and/or chronic stress results in an imbalance in the production and accumulation of free radicals (reactive oxygen species) which can induce oxidative stress. Oxidative stress causes base damage and DNA strand breaks resulting in apoptosis and necrosis. Therefore, oxidative stress response and DNA strand repair can preserve genome integrity and normal mechanisms of cellular signaling under stress.

In conclusion, despite the complexity of the agro-ecological and climatic conditions of Africa, domestic goats occur across the continent. The results presented here show at least two genomic ancestries in African goats, and that genetic divergence and genomic plasticity is the driver of the successful integration of the species into African environments. These findings are significant in the context of improving livestock productivity in the continent in view of the projected consequences of climate change on biodiversity. A complete characterization of African indigenous goat’s unique genomic variation and adaptation can inform the formulation and design of breeding programs that promote the long-term sustainable goat productivity.

## Materials and methods

### DNA samples and sequencing

**Figure 5:**
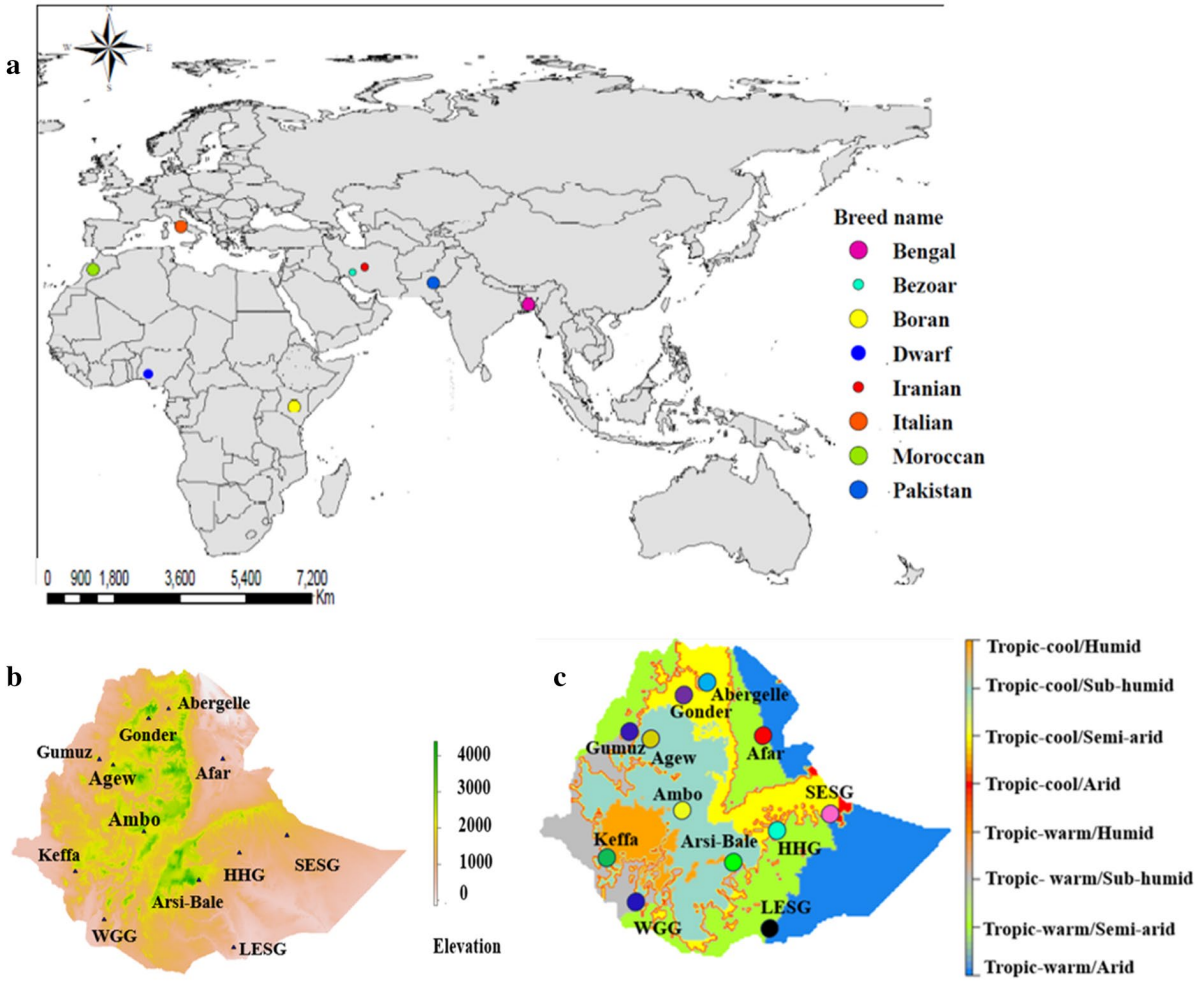
Map of the study areas (**a**) The geographic location of the African, South Asian, Middle Eastern, European, and Bezoar goat populations, (**b**) the geographic distribution of Ethiopian goat populations based on elevation, and (**c**) Agro-ecological zones (Sources: Own processed maps using global data set in ArcGIS environment version 10.8).

Twelve indigenous Ethiopian goat populations (Supplementary Table 1; Fig. 5b,c) that were previously described in Tarekegn ^11,15^ were used in this study. No ethics permissions were required. DNA samples of five individuals per population were selected at random and whole genome sequenced in Novogene, China (https://en.novogene.com/services/reserachservices/genome-sequencing/whole-genome-sequencing-wgs/). Whole-genome sequencing was performed on an Illumina NovaSeq 6000 Platform (Illumina, San Diego, CA, USA) and 150 bp of paired-end reads at a target coverage depth of 10× were generated. The quality of the sequences was assessed with FASTQC v0.11.5^80^ and three samples failed quality control (Supplementary Table S1). For comparative genome analysis and referencing, whole-genome sequences of 67 individuals of the east (Kenya, Boran goat), west (Nigeria, Dwarf goat) and north-west (Morocco) African (n = 20), South Asian (Pakistan and Bangladesh; n = 17), Middle East (Iran, n = 10) and European (Italy; n = 10) domestic goats, and 10 of wild Bezoar goats obtained from public databases (https://www.goatgenome.org/vargoats.html) were included in the study (Supplementary Table S1; Fig. 5). The wild Bezoar goats, an extant species found in western Asia from Turkey to Pakistan is the presumed ancestor of modern-day domestic goats^2^. All clean reads were aligned to the *C*. *hircus* reference genome (ARS1; GenBank accession number GCA_001704415.1) using the BWA tool v0.7.17^81^. The alignment files in SAM format were converted to BAM format with SAMtools^82^.

We applied the GATK v3.8 workflow^83^ for variant calling and discovery. SAMTools was used to sort and index the alignment files and duplicate reads were purged using PICARD v2.18.2 (https://broadinstitute.github.io/picard/). Base Quality Score Recalibration was used to create recalibrated BAM files from the recalibrated table. Variant Quality Score Recalibration was performed using VariantRecalibrator in GATK with the “knownSites” set to the *C. hircus* dbSNP reference panel (https://e99.ensembl.org/capra_hircus). Individual VCF files were called using the HaplotypeCaller in GATK followed by GenotypeGVCFs to generate consolidated GVCF files that contained the raw SNPs and Indels. Variant refinement was performed using variantRecalibrator in GATK. Variant call annotations such as Read Depth, Quality of Depth, Fisher Strand Test, Mapping Quality Score, Mapping Quality Rank Sum Test, Read Position Rank Sum Test Statistic, StrandOddsRatio Test, mode SNP and the VQSRTranchesSNP90 to 100 were used to produce recalibrated SNPs. Using ApplyRecalibration, a tranche sensitivity threshold of 99% was used to generate filtered variants, and post-processing using SelectVariants was conducted to remove variants that failed the GATK filtering parameters. In this step, a total of 26,990,415 and 56,648,064 markers remained which, included autosomal and sex chromosomes for Ethiopian, and other (African, Eurasian, and wild Bezoar) goats, respectively. Further, quality control using the command line ‘SelectVariant’ and ‘restrictAlleles’ were used to remove sex chromosomes and multiallelic SNPs, respectively. Finally, the subsequent analyses were performed using 24,759,579 and 42,728,409 autosomal biallelic markers found across 57 and 124 individuals from 12 and 25 populations, respectively (Table 1).

### Genome-wide genetic diversity and dynamics

Within-population variation was determined by estimating observed (H_O_) and expected (H_E_) heterozygosity, nucleotide diversity (π), within-population genetic distance (D_ST_), and two inbreeding coefficients (F_HOM_ and F_RoH_). The H_O_, H_E_ and π were estimated with VCFTools v0.1.15^84^. To compute π, a 20 kb window size and a sliding step of 10 kb was used. For each population and group of populations, SNP density was also calculated with VCFtools v0.1.15 with the command line “–SNPdensity1000” and its mean and standard deviation was computed with R v4.1.0^85^. The D_ST_ was calculated using the command line “–genome” in PLINK v1.9^86^. The genetic distance between all individuals within a population was then calculated as D = 1 − D_ST_.

Runs of homozygosity (RoH) were identified using PLINK v1.9 with the following command line parameters following Guo^87^, ‘–homozyg-window kb 5000 -chr-set 29 –homozyg-window-snp 50 –homozyg window-het 1 –homozyg-snp 10 –homozyg-kb 100 –homozyg-density 10 –homozyg-gap 100’. The mean number and length of RoH were determined at four-genome length categories: 0.1–0.25, > 0.25–0.5, > 0.5–1, and > 1 Mb. The RoH-based inbreeding coefficient (F_RoH_) was computed as the average genome covered by RoH divided by the length of the ARS1 goat reference genome assembly.

Demographic dynamics were investigated by assessing pairwise LD between all pairs of autosomal biallelic variants (SNPs) over genomic distance through the correlation coefficient (*r*^2^) with PLINK v1.9. Historic demographic dynamics were assessed by investigating the trends in effective population size (N_e_) over generation time (up to 1000 generations ago) as described in Ahbara et al.^22^.

### Genetic structure and relationships

Variation between populations was investigated at two levels: (1) for Ethiopian goats only and (2) for the combined dataset of Ethiopian and non-Ethiopian goat populations. The variation was explored and visualized with principal component analysis (PCA), ADMIXTURE tool, and Neighbour-Joining (NJ) phylogenetic tree. Only autosomal loci were used and those in LD were pruned with the –indep-pairwise function in PLINK1.9 using a window size of 50 kb, and step size of 10 kb, and r^2^ 0.01. Out of the 24,759,579 autosomal SNPs in Ethiopian goats and the 42,728,409 autosomal SNPs in the overall dataset, 7,650,233 and 18,466,404 SNPs, respectively passed the LD filter and were used to assess population structure and relationships.

PCA was performed with PLINK v1.9 running the –pca command and the results were visualised by plotting the first two PCs using the tidyverse package of R v4.1.0^85^. The F_ST_-distance matrices between populations were generated using Vcftools v0.1.15^84^ and were then used to reconstruct an NJ phylogenetic tree with R software. The unsupervised block relaxation algorithm implemented in ADMIXTURE v1.3^88^ was used to determine the proportion of shared genome ancestry between populations. A five-fold cross-validation procedure following Lawal et al.^89^ was used to determine the optimal number of genome clusters/groups (K) and the proportion of shared genome ancestry.

### Genome annotations

To investigate whether population divergence revealed by cluster analysis, could be the outcome of adaptive radiation, we combined the results of PCA (Fig. 1a) with the topographic (Fig. 5b) and agro-eco-climatic distribution (Fig. 5c) of Ethiopian goats and selected Afar, Arsi-Bale and Keffa goats as the proxies to investigate selection signatures resulting from genomic divergence.

We used 19.28 million autosomal biallelic SNPs to run three approaches, pooled heterozygosity (H_P_)^90^, fixation index (F_ST_)^91^, and cross-population extended haplotype homozygosity (XP-EHH)^92^. A sliding window of 100 kb size with 50 kb sliding step was applied for H_P_ and F_ST_ tests. To avoid spurious signals, sliding windows with < 10 SNPs were discarded. The expected heterozygosity (H_P_) within each window was calculated using an in-house R script. For each SNP, the number of reads corresponding to the most (n_MAJ_) and least (n_MIN_) abundant alleles for each window in each population were used to calculate the H_p_ score as: H_P_ = 2∑n_MAJ_∑n_MIN_/(∑n_MAJ_ + ∑n_MIN_)^2^; where, ∑n_MAJ_ and ∑n_MIN_ are the sums of n_MAJ_ and n_MIN_ for all the SNPs in the windows. Individual H_p_ values were then Z-transformed using the formulae ZH_p_ = (H_p_ − μH_p_)/σH_p_. The F_ST_ value for each SNP between the three populations (Arsi-Bale, Afar, Keffa) was calculated using VCFtools (v0.1.15) to assess genetic differentiation. The F_ST_ value was then Z-transformed into ZF_ST_ with the formula: ZF_ST_ = (F_ST_ − μF_ST_)/σF_ST_. Putative selection targets were extracted from the extreme tail ends of the empirical distributions by applying a ZH_P_ score <  − 3.9 and the corresponding ZF_ST_ value > 4 as the cut-off thresholds. We compared the extended haplotype homozygosity (EHH) among the three populations (Arsi-Bale, Afar, Keffa) using the XP-EHH statistic estimated with the REHH package^93^ in R. The unstandardized XP-EHH statistics were standardized using their means and variances. We estimated the p-values of the SNPs using the standard normal distribution following Sabeti et al.^92^. Regions falling within the top 0.001% of the empirical distribution or above XP-EHH score ≥ 5 were identified and taken to be the candidate selection sweep regions. All genes that either completely or partially overlapped with the candidate selection sweep regions were identified based on the ARS1 *C. hircus* reference genome gene annotations with the Ensembl BioMart (http://www.biomart.org) tool.

For functional classification, we retrieved genes within each candidate selective sweep region using Ensembl BioMart version 104. These gene lists were used for gene ontology (GO) (http://geneontology.org/) and KEGG (Kyoto encyclopedia of genes and genomes) (http://www.genome.jp/kegg/pathway.html) analyses implemented in DAVID version 6.8 (http://david.ncifcrf.gov/)^94^. We used all RefSeq genes in the *C. hircus* genome as background. Overrepresented gene clusters were identified by Fisher’s exact tests (p < 0.05) and biological processes, cellular components and molecular functions were used as GO term categories with a significance level of *p-value* < 0.05.

### Supplementary Information

Supplementary Figures.

Supplementary Tables.

## Data Availability

The data generated herein have been deposited in NCBI under Sequence Read Archive (SRA) accession number SRP4642793^95^.
