# Supplementary material for: Anthropogenic events and responses to environmental stress are shaping the genomes of Ethiopian indigenous goats
Source: Sci Rep. 2024 Jun 28;14:14908. doi: 10.1038/s41598-024-65303-x (PMC11213886; doi:10.1038/s41598-024-65303-x)
Supplement: Supplementary file 1 — Supplementary Figures. [file 41598_2024_65303_MOESM1_ESM.docx]

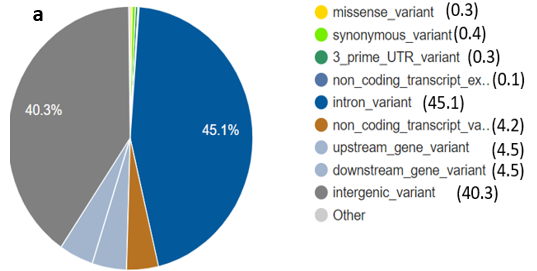

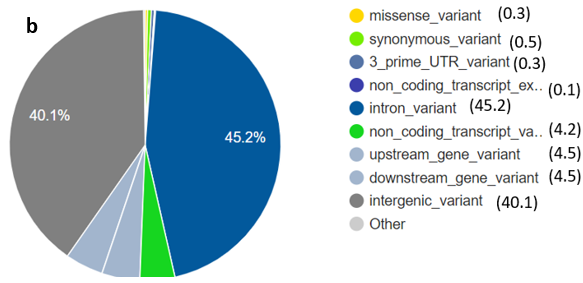


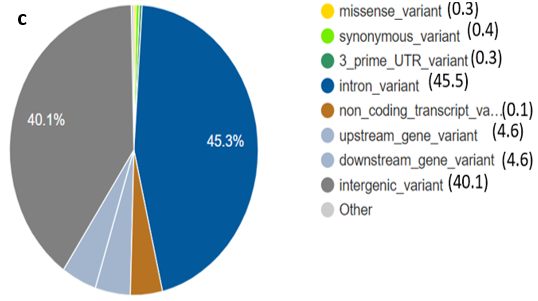

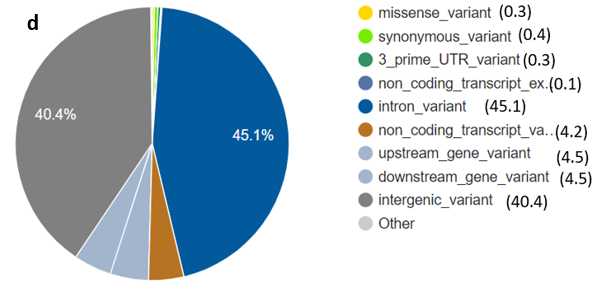


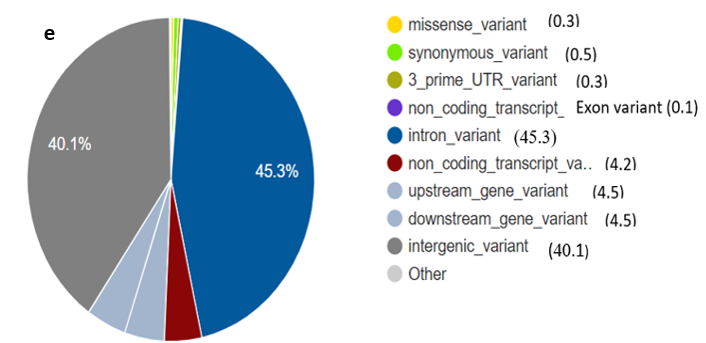


**Fig. S1:** Consequences (all). (**a**) African, (**b**) European (Italian), (**c**) Asian (South Asian and Middle East), (**d**) Bezoar, and (**e**) Ethiopian goat populations.


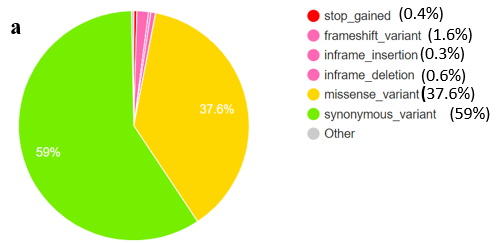

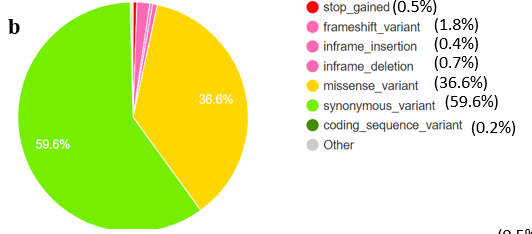


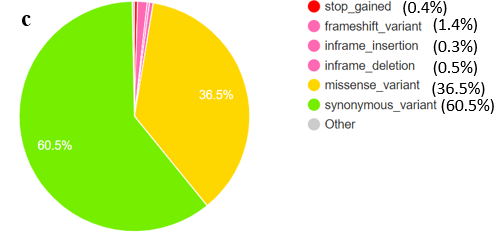

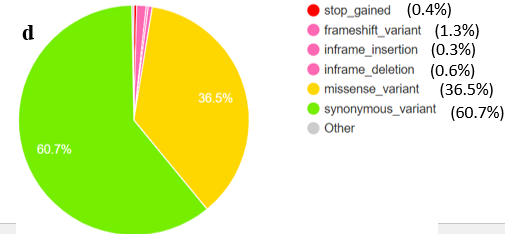


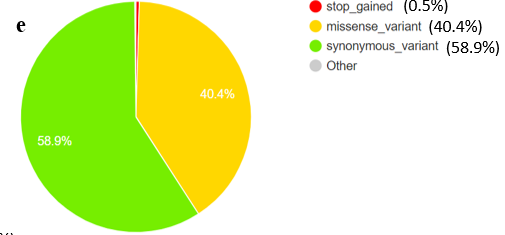


Fig. S2: Coding consequences. (**a**) African, (**b**) European (Italian), (**c)** Asian (South Asian and Middle East), (**d**) Bezoar, and (**e**) Ethiopian goat populations.


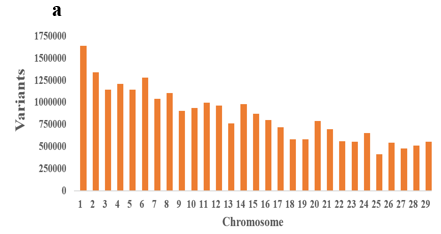

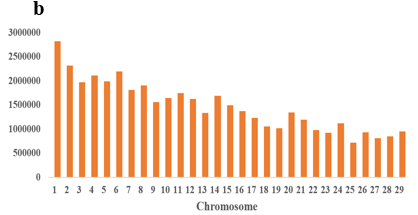


**Fig. S3:** Average variant by chromosome distribution. (A) Ethiopians, (B) other African, Eurasian and wild Bezoar goat populations.


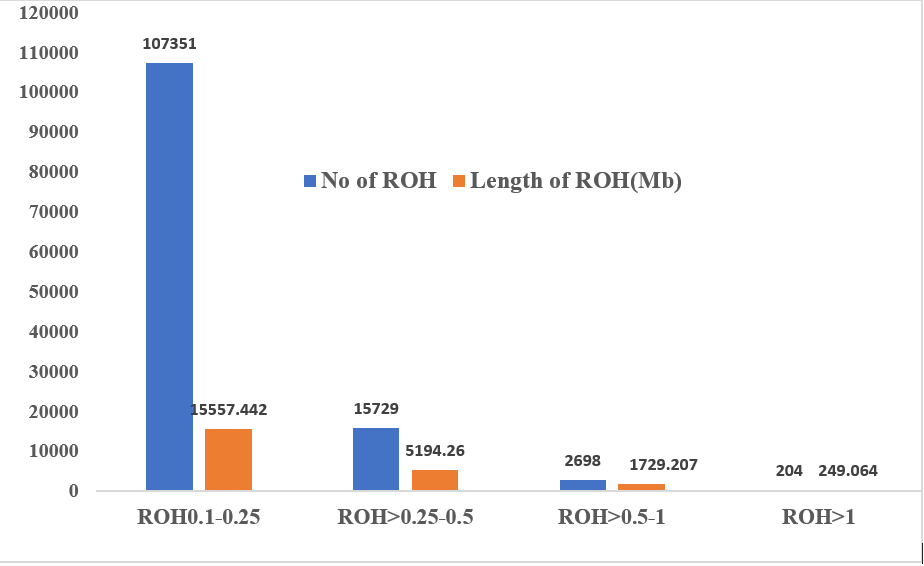


**Fig. S4**: Genome-wide distribution of the number and length of ROH at different length category across Ethiopian and other African, Eurasian, and wild Bezoar goats. The trend is similar for all goat populations. This runs of homozygosity was identified using PLINK software v1.9 with the command:

-homozyg-kb 100: length in Kb of the sliding window

-homozyg-window-snp 50: Number of SNPs that the sliding window must have),

-chr-set 29: To indicate goat chromosome number

-homozyg-snp 10: Minimum number of SNPs that a ROH is required to have

-homozyg-density 10: required minimum density to consider a ROH or 1 SNP in 10kb)

-homozyg-gap 1000: Length in Kb between two SNPs,

-homozyg-window-threshold 0.05: Proportion of overlapping windows that must be called homozygous to define a given SNP as in a “homozygous” segment

-homozyg-window-missing 2: Number of missing calls allowed in a window

-homozyg-window-het 1: Number of heterozygous SNP allowed in a window. PLINK allows the setting of different variable number of heterozygous SNPs per window, with a default value of 1 heterozygous genotype per ROH, to tolerate genotyping calling errors.


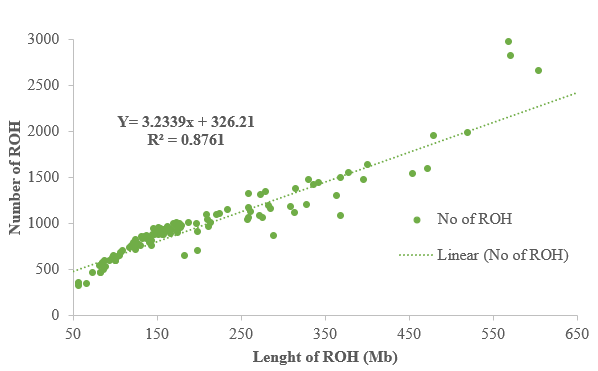


**Fig. S5**: The pattern of the Run of homozygosity and the correlation between the total number of ROH and its length across Ethiopian and other African, Eurasian, and wild Bezoar goat populations.


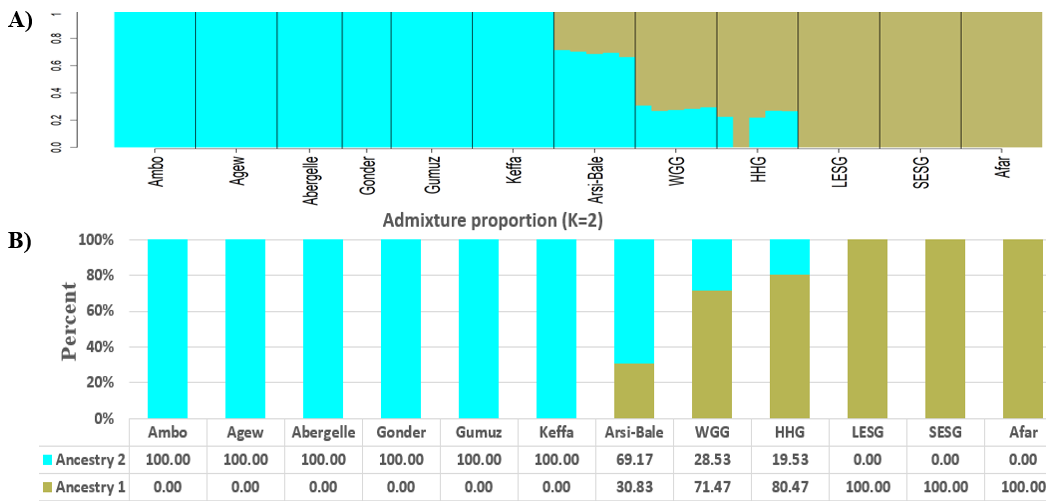


**b**

**a**


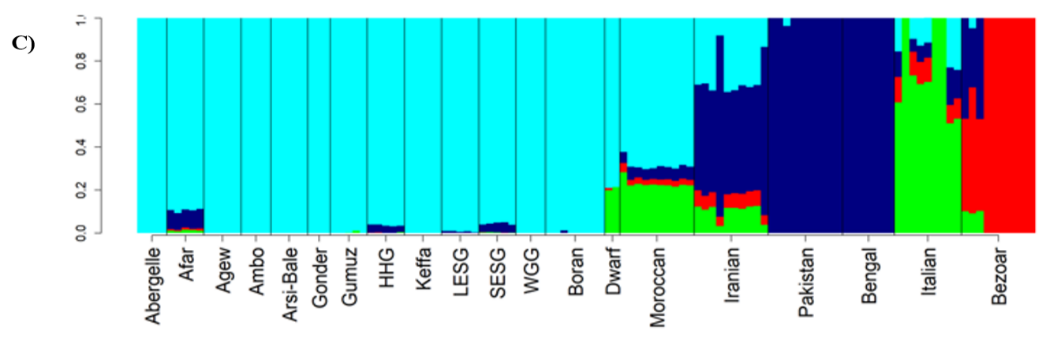


**c**


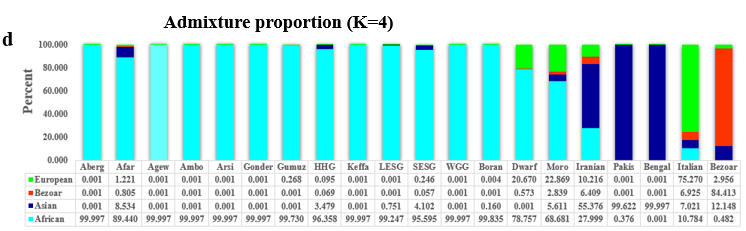


**Fig. S6:** Population ADMIXTURE plot. (**a**) ADMIXTURE plot of the Ethiopian goats at K=2 and (**b**) ADMIXTURE proportion for the Ethiopian goats, (**c**) ADMIXTURE plot of the African, European, Asian (South Asian, Middle East), and wild Bezoar goats at K=4 and (**d**) it’s ADMIXTURE proportion.
